# Advances in Non-CPAP Management of Obstructive Sleep Apnea: Spotlight on Pharmacological Therapies

**DOI:** 10.3390/jpm16020105

**Published:** 2026-02-10

**Authors:** Matteo Siciliano, Martina de Scisciolo, Antonio Fratini, Costanza Sottani, Federico Giordani, Valerio Brunetti

**Affiliations:** 1 Independent Researcher, 00168 Rome, Italy; mat.sic89@gmail.com; 2 Department of Neurology, Catholic University of Sacred Heart, 00168 Rome, Italy; martina.descisciolo01@icatt.it; 3 Unità Operativa Semplice di Malattie dell’Apparato Respiratorio, Unità Operativa Complessa di Medicina Generale, Cristo Re Hospital, 00167 Rome, Italy; antoniofratini2@gmail.com; 4 Neurology Unit, Ospedale San Pietro Fatebenefratelli, 00189 Rome, Italy; sottani.costanza@fbfrm.it; 5 Department of Pulmonology, Catholic University of Sacred Heart, 00167 Rome, Italy; federico.giordani01@icatt.it; 6 UOC di Neurologia, Dipartimento di Neuroscienze, Organi di Senso e Torace, Fondazione Policlinico Universitario A. Gemelli IRCCS, 00168 Rome, Italy

**Keywords:** obstructive sleep apnea, excessive daytime sleepiness, non-CPAP therapies, pharmacological treatment, OSA endotypes, wake-promoting agents

## Abstract

Obstructive sleep apnea (OSA) is a highly prevalent sleep-related breathing disorder associated with significant cardiometabolic morbidity, impaired neurocognitive functioning, daytime sleepiness, and reduced quality of life. Although continuous positive airway pressure (CPAP) therapy remains the cornerstone of treatment for moderate-to-severe OSA, long-term adherence is frequently suboptimal, and a substantial proportion of patients experience residual symptoms despite adequate therapy. These limitations have driven increasing interest in non-CPAP treatment strategies and, more recently, in pharmacological approaches tailored to specific OSA pathophysiological mechanisms. This narrative review provides an updated overview of non-CPAP therapies for OSA, including oral appliances, surgical interventions, positional therapy, hypoglossal nerve stimulation, and behavioral strategies, with a particular focus on emerging and established pharmacological treatment and their role in endotype/phenotype-guided management of OSA. Overall, the expanding pharmacological landscape of OSA reflects a paradigm shift toward personalized, multimodal management. Integrating non-CPAP and pharmacological therapies with patient-specific pathophysiology may improve symptom control, adherence, and long-term outcomes in OSA.

## 1. Introduction

Obstructive sleep apnea (OSA) is the most common sleep disordered breathing, whose incidence and prevalence are continuously expanding. It is characterized by partial or complete collapse of the airways during sleep, leading to numerous pathophysiological consequences, initiated by recurrent episodes of intermittent hypoxia, intrathoracic pressure swings, and sleep fragmentation. It is associated with numerous comorbidities, especially cardiovascular issues and even increased mortality [1]. Prevalence estimates vary widely based on population, diagnostic criteria, and measurement methods. Nevertheless OSA is estimated to affect 1 billion individuals aged 30–69 years worldwide, with approximately 425 million experiencing moderate-to-severe disease [2]. OSA has long been considered a male-predominant condition, with studies showing a higher prevalence in males compared to females. However, more recent research highlights less pronounced difference in male-to-female patient ratio (1.5:1), female patients being up to 40–50% of presentations at sleep clinics [3].

Pathogenesis of OSA can be multi-factorial, involving anatomical and non-anatomical factors well documented in the PALM scale proposed by Eckert et al.: pharyngeal critical closing pressure (Pcrit, P), decreased respiratory arousal threshold (arousal threshold, A), increased loop gain (loop gain, L), and upper airway dilator muscle activity (muscle responsiveness, M) are the main mechanisms contributing to the pathogenesis [4]. This pathophysiological framework is crucial for tailoring treatment to the individual patient.

Positive airway pressure (PAP) treatment, according to AASM guideline [5] is the first-line choice therapy for patients with moderate-to-severe OSA, creating a “pneumatic splint” for the upper airways avoiding their collapse. Its effectiveness is evidence-based, since PAP treatment in adults with OSA significantly decreases disease severity, sleepiness, blood pressure, and the risk for motor vehicle accidents, and improves sleep-related quality of life [6]. Compliance with PAP therapy has always been a debated topic and the biggest problem to deal with in daily clinical practice. Use of the device for four or more hours per night on 70% of nights during a consecutive 30-day period is considered an adequate use and, in addition, the minimum use for reimbursement according to Centers for Medicare and Medicaid Services (CMS) criteria [7]. Patients’ perception and acceptance of PAP therapy play a crucial role in achieving acceptable adherence. Adherence rates range from 40 to 85%, and many factors may affect it: sociodemographic characteristics, disease severity, psychosocial factors and therapy side effects [8]. Although a variety of non-continuous positive airway pressure (CPAP) options are currently available, these approaches should generally be considered complementary or second-line alternatives, with selection guided by the patient’s phenotype and underlying pathophysiological traits.

The recent characterization of various clinical and pathophysiological phenotypes of OSA, and moreover data related to limited adherence, has opened ways for alternative treatments to PAP, resulting in a more personalized approach. Recently, the European Respiratory Society (ERS) has presented a guideline on non-CPAP therapies for OSA, updated an older statement on non-CPAP treatment [9].

## 2. Non-CPAP Therapy in OSA: An Overview

OSA management is evolving beyond CPAP treatment, with increasing attention to individualized, phenotype-driven approaches (anatomy, collapsibility, loop gain, arousal threshold) to improve effectiveness and adherence. This overview briefly summarizes current evidence on non-CPAP therapies, including oral appliances, upper-airway surgery, positional and behavioral strategies, and hypoglossal nerve stimulation (HNS) [9,10,11]. Pharmacological approaches will be discussed in detail later in this review.

Non-CPAP therapies should be considered when CPAP fails, is not tolerated, or is declined by the patient [9]. Patient selection should integrate clinical phenotype, anatomical characteristics, comorbidities, disease severity and underlying pathophysiological traits to optimize outcomes within a personalized treatment network. Long-term CPAP adherence remains a challenge, and up to 50% of patients discontinue use within the first year [12].

Oral appliances, particularly custom-made, titratable mandibular advancement devices (MADs), are the most established non-CPAP therapy for OSA. Randomized trials and meta-analyses show that MADs reduce the apnea–hypopnea index (AHI) and improve daytime symptoms compared to placebo, although efficacy is generally lower than CPAP [13,14,15,16]. MADs are recommended for patients with mild to moderate OSA or those who are CPAP-intolerant and require dental/occlusal follow-up for titration and monitoring of side effects [9]. Despite smaller reductions in AHI, MADs may provide comparable improvements in sleepiness and blood pressure, likely supported by higher adherence in many patients [12,17]. Selected patients with severe OSA may also benefit, with complete resolution reported in up to one third of cases [18]. Treatment response is enhanced in individuals with favorable craniofacial anatomy and mild positional dependency [12].

Surgical treatment may be appropriate in carefully selected patients, particularly those with unfavorable upper-airway anatomy or intolerance to CPAP. Surgical approaches include uvulopalatopharyngoplasty (UPPP), expansion sphincter pharyngoplasty (ESP), and maxillo-mandibular advancement (MMA). UPPP is a palatal surgical procedure designed to enlarge the retropalatal airway by removing the uvula and redundant portions of the soft palate, often combined with tonsillectomy. Although widely performed, UPPP is associated with variable efficacy on AHI reduction and a non-negligible risk of post-operative morbidity, limiting its role as a stand-alone treatment for obstructive sleep apnea [10].

ESP is a reconstructive palatal procedure that targets lateral pharyngeal wall collapse by repositioning the palatopharyngeus muscle, thereby enlarging and stabilizing the retropalatal airway. In appropriately selected patients, particularly those with predominant lateral wall collapse on drug-induced sleep endoscopy (DISE), ESP is associated with moderate reductions in the AHI and improvements in symptoms and quality of life, with lower morbidity compared with traditional resective palatal surgeries [19].

Compared with palatal procedures, MMA generally provides the greatest efficacy, although it is reserved for selected anatomical phenotypes [20]. MMA is a craniofacial surgical procedure that enlarges the upper airway by anteriorly repositioning the maxilla and mandible, thereby increasing pharyngeal airway volume and reducing collapsibility. Although it represents the most effective surgical treatment for OSA, with high response and cure rates in carefully selected patients, particularly those with craniofacial abnormalities, its invasive and potentially morbid nature limits its use to patients with severe disease or failure/intolerance of less invasive therapies [18]. Therefore, MMA should be considered in selected patients with severe OSA and craniofacial abnormalities; UPPP alone is not routinely recommended [9].

Surgical success depends strongly on patient selection and should be considered in patients with anatomical obstruction unresponsive to conservative measures [12]. Multilevel surgery based on DISE improves outcomes [21].

Hypoglossal nerve stimulation (HNS) is a second-line option for moderate-to-severe OSA in non-obese, CPAP-intolerant patients. It significantly improves AHI and sleep-related symptoms. The American Academy of Otolaryngology–Head and Neck Surgery supports its use as a safe and effective alternative in selected cases. Transcutaneous stimulation and newer devices are under evaluation and may expand indications [18]. HNS is supported in patients with moderate-to-severe OSA, BMI < 35 kg/m^2^, and no complete concentric collapse on DISE [9]. HNS provides sustained benefit in cohorts meeting STAR-like criteria (e.g., AHI approximately 20–50, BMI lower, no complete concentric collapse on DISE), with 5-year durability; cost and candidacy constraints apply [13,14]. However, real-world implementation may be limited by high upfront costs, reimbursement policies, availability in specialized centers, and strict candidacy requirements, which restrict access to a subset of eligible patients.

Positional therapy benefits positional OSA but is generally less effective than CPAP; adherence variability limits real-world impact [13]. Vibratory feedback devices improve adherence compared to older mechanical techniques, though efficacy is generally lower than CPAP. It is best reserved for mild to moderate OSA [22]. Positional therapy may also be part of a multimodal strategy, and supine avoidance remains an effective but underused adjunct [18], with supine-related OSA patient being the best candidates for this approach [12]. In clinical practice, positional interventions are often most valuable when integrated into a personalized, multimodal treatment plan, either as an adjunct to CPAP or combined with other non-CPAP therapies to enhance overall effectiveness and adherence.

Behavioral therapy such as weight loss and exercise improves OSA severity and cardiometabolic risk and should be part of all care plans; avoidance of alcohol/sedatives near bedtime is advised [13,14,15]. Even a mild weight loss can significantly reduce AHI [23].

## 3. Pharmacological Therapy for OSA

Pharmacotherapy for OSA is an emerging field. To date, no medication is approved as a universal replacement for CPAP across the overall OSA population; however, in the United States tirzepatide is labeled for the treatment of moderate-to-severe OSA in adults with obesity [24]. Other drugs are labeled in OSA only for the management of excessive daytime sleepiness (e.g., modafinil, armodafinil, solriamfetol; and pitolisant in the European Union), rather than for treating the underlying airway obstruction.

**Figure 1:**
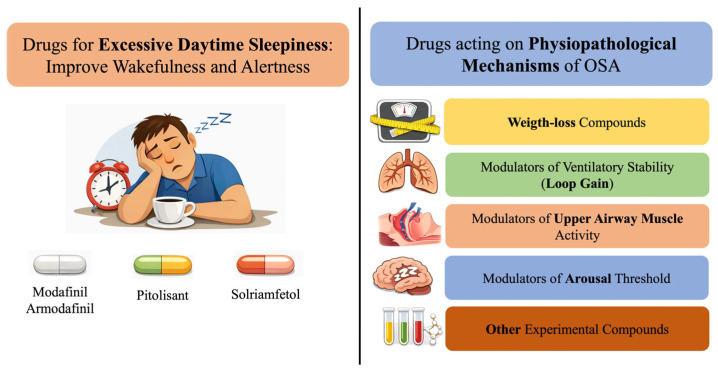
**Current Pharmacological Treatments for OSA.** Wake-promoting agents (**left**) provide symptomatic relief of residual excessive daytime sleepiness and do not treat the underlying airway obstruction. Agents targeting OSA pathophysiological mechanisms (**right**) are intended to reduce OSA severity (for example AHI and hypoxic burden) and represent potential disease modifying approaches, although most remain investigational and are not substitutes for CPAP.

At present, pharmacological approaches may complement established therapies or be considered in selected patient subgroups. These treatments can be broadly divided into two main categories (Figure 1): (1) agents designed to directly reduce the apnea–hypopnea index (AHI) by targeting specific underlying pathophysiological mechanisms [25]; and (2) wake-promoting agents (WPAs), including modafinil/armodafinil, pitolisant, and solriamfetol, which are indicated for the management of residual excessive daytime sleepiness (rEDS) and are typically prescribed alongside CPAP in patients who remain symptomatic despite effective nocturnal therapy [26].

### 3.1. Obstructive Sleep Apnea Endotypes: Distinct Pathophysiological Pathways

Four key pathophysiological pathways have been identified as underlying mechanisms of obstructive sleep apnea (OSA): anatomical factors, such as a narrow and collapsible upper airway; impaired responsiveness of upper airway dilator muscles; a low respiratory arousal threshold; and high loop gain, reflecting an overly sensitive ventilatory control system [27]. Anatomical vulnerability is primarily addressed through pharmacological agents targeting obesity, most notably GLP-1 receptor agonists and dual GIP/GLP-1 receptor agonists [28]. Strategies aimed at enhancing upper airway muscle tone during sleep include noradrenergic–antimuscarinic combinations and potassium channel blockers, which seek to improve neuromuscular stability of the upper airway [29].

High loop gain is targeted by carbonic anhydrase inhibitors, such as zonisamide and sulthiame, which currently represent the main pharmacological approach to stabilizing ventilatory control by dampening chemosensitivity [30]. Finally, pharmacological modulation of the arousal threshold has largely relied on sedative agents, including benzodiazepines and Z-drugs; by increasing the arousal threshold, these medications reduce the propensity for premature nocturnal awakenings, although their clinical utility remains limited by safety concerns [31].

### 3.2. Weight Loss Medications

Obesity is a major risk factor for OSA, as excess adiposity increases upper-airway collapsibility through mechanical loading of pharyngeal structures and obesity-related reductions in lung volume, while also promoting systemic inflammation and impaired neuromuscular control of the upper airway [32]. Anti-obesity pharmacotherapy has emerged as a relevant adjunct in OSA, particularly when disease severity is largely obesity-driven. Crucially, improvements in OSA severity with these agents are driven predominantly by weight loss, rather than by a direct, weight-independent effect on upper-airway physiology. Although weight-loss pharmacotherapy can lead to clinically meaningful improvements in OSA severity, treatment response is variable across individuals and closely depends on the magnitude and durability of weight reduction. Moreover, long-term adherence to anti-obesity medications and the sustainability of their effects over time remain important limitations that may affect real-world effectiveness.

To date, the anti-obesity agents for which randomized controlled trial (RCT) data in OSA are available include: liraglutide, a GLP-1 receptor agonist [33]; tirzepatide, a dual GIP/GLP-1 receptor agonist [34]; and phentermine–topiramate extended release, a sympathomimetic anorectic combined with an antiepileptic agent [35]. In addition, armodafinil, a wake-promoting agent, was evaluated in a 6-month double-blind, parallel-group trial [36] primarily because appetite suppression is a known side effect; however, it did not result in any reduction in AHI nor improvement in daytime sleepiness, limiting its relevance as an anti-obesity strategy in OSA.

Among currently available anti-obesity agents, tirzepatide has demonstrated the greatest efficacy in reducing OSA severity; it also induced the largest weight loss, significantly reduced hypoxic burden, and lowered high-sensitivity C-reactive protein (hs-CRP), and it was the only agent to improve quality of life, as assessed by the Patient-Reported Outcomes Measurement Information System (PROMIS) Sleep-Related Impairment and Sleep Disturbance scales [37,38]. The phase 3 SURMOUNT-OSA trials evaluated tirzepatide in adults with moderate-to-severe OSA and obesity [34]. In Trial 1, involving participants not using PAP, tirzepatide reduced the AHI by 25.3 events per hour at 52 weeks, compared to 5.3 with placebo, a 50.7% reduction from baseline (*p* < 0.001). In Trial 2, among PAP users, the AHI reduction was 29.3 vs. 5.5 events per hour, reflecting a 58.7% reduction (*p* < 0.001). These reductions were clinically meaningful, meeting the commonly accepted threshold of 50% AHI improvement [34]. Tirzepatide also improved key secondary outcomes, including body weight, hypoxic burden, inflammation (hsCRP), systolic blood pressure, and sleep-related quality of life. The most common adverse events were mild to moderate gastrointestinal symptoms, mainly during dose escalation, consistent with the known safety profile of GLP-1-based therapies [34]. The US Food and Drug Administration (FDA) recently approved tirzepatide for moderate-to-severe OSA in adults with comorbid obesity [24].

Beyond tirzepatide, liraglutide and phentermine–topiramate extended release have been shown to produce significant weight loss with modest, but potentially clinically meaningful, reductions in AHI in cohorts selected for overweight/obesity [37]. In the SCALE Sleep Apnea randomized trial, liraglutide 3.0 mg was associated with a greater reduction in AHI than placebo in obese patients with moderate-to-severe OSA, irrespective of CPAP use [33]. By contrast, phentermine–topiramate XR was associated with a higher burden of adverse effects, including elevated heart rate, dry mouth, and dysgeusia [37].

### 3.3. Modulators of Upper Airway Muscle Activity

Pharmacological strategies targeting upper-airway neuromuscular control have emerged as one of the most promising non-weight-based approaches for OSA, as they aim to enhance pharyngeal dilator muscle activity during sleep and thereby reduce airway collapsibility independently of anatomical change. Patients with impaired pharyngeal dilator muscle responsiveness may be particularly well suited to noradrenergic–antimuscarinic combination therapy. Noradrenergic reuptake inhibitors (e.g., atomoxetine, reboxetine) facilitate activation of pharyngeal dilator motoneurons, particularly in non-REM sleep [39]. Antimuscarinic agents (e.g., oxybutynin, hyoscine butylbromide) are used in combination to counter muscarinic inhibition of hypoglossal motoneurons, a mechanism considered especially relevant during REM sleep when cholinergic influences contribute to hypotonia [40]. Initial proof of concept came from randomized crossover data showing that single-night atomoxetine–oxybutynin can produce large reductions in AHI (approximately 60–70%), alongside improvements in oxygenation and genioglossus responsiveness, supporting a direct neuromuscular mechanism rather than a sedative or arousal-mediated effect [41]. Building on this, the MARIPOSA phase II trial evaluated AD109 (atomoxetine plus aroxybutynin) administered nightly for four weeks and reported placebo-adjusted AHI reductions of roughly 43–47% with parallel improvements in hypoxic burden and oxygen desaturation indices in the absence of weight loss, reinforcing the plausibility of a weight-independent effect [42]. Notably, AD109 remains an investigational combination that is not currently labeled for routine clinical use, and its clinical role will depend on the results of larger, longer phase III programs and regulatory review.

At the same time, the current evidence base warrants a more cautious interpretation. Across trials of noradrenergic–antimuscarinic (and related anticholinergic) regimens, treatment durations have ranged from a single night to about one month, with more than half of studies being single-night experiments, and adverse events were frequently underreported, with several studies providing no safety data [43]. Even within the available safety reporting, adverse effects have included dry mouth, nausea, and urinary hesitancy, as well as sleep architecture disruption, most notably a marked reduction in REM sleep percentage, which is a potentially important concern given REM-related vulnerability in many OSA patients [43]. Moreover, pooled analyses suggest that these combinations may lower the arousal threshold versus placebo only when the arousal threshold is quantified as “% of eupnea”, and they have not demonstrated consistent symptomatic benefit versus placebo on patient-centered outcomes such as the Karolinska Sleepiness Scale (KSS), Epworth Sleepiness Scale (ESS), or sleep quality visual analog scales [43]. Collectively, these limitations, such as short exposure, incomplete safety characterization, REM suppression, and uncertain symptomatic impact, help explain why these regimens remain investigational despite robust short-term physiological effects.

Serotonergic approaches have generally been less consistent and often limited by adverse effects [12,40]. More recent arousal-modulating combinations, including trazodone-based strategies, have shown intriguing short-term signals in selected cohorts [44,45,46], but given the still limited duration of exposure and the priority need for robust tolerability and longitudinal outcomes, they currently remain best framed as exploratory rather than practice-changing.

Overall, noradrenergic–antimuscarinic combinations provide strong short-term proof that targeting non-anatomical mechanisms can substantially reduce OSA severity in selected patients. However, the translational gap to clinical adoption remains driven by the brevity of available trials, underreported and incompletely characterized adverse effects, potential sleep-architecture trade-offs, and the lack of consistent improvements in patient-reported symptoms, underscoring the need for longer, outcomes-focused randomized studies before these therapies can be positioned beyond a phenotype- and endotype-driven investigational framework [43].

### 3.4. Modulators of Ventilatory Stability (Loop Gain)

Carbonic anhydrase inhibitors (CAIs) represent an endotype-targeted pharmacological approach to OSA by primarily reducing ventilatory control instability. However, no CAI is currently labeled for OSA, and because the available evidence is largely derived from small, short-duration studies, with uncertain long-term outcomes and incompletely characterized effects on common comorbidities, the 2021 ERS guideline on non-CPAP therapies conditionally recommends CAIs only within the context of randomized controlled trials [9]. Among agents studied, sulthiame has shown short-term reductions in OSA severity in the order of roughly one third to 40% and improvements in nocturnal oxygenation and sleep fragmentation; in a 4-week randomized, double-blind, placebo-controlled trial in CPAP-intolerant patients, mean AHI fell by 35–40%, and a ≥ 50% reduction was achieved in up to 40% of participants at 400 mg, albeit typically without normalization of AHI, with paresthesias and headaches occurring more frequently at the higher dose [47]. Acetazolamide has likewise demonstrated meaningful short-term reductions in AHI and loop gain, with the additional observation of blood pressure lowering in hypertensive OSA cohorts; tolerability is generally acceptable in short trials, with class-typical adverse effects including paresthesia, dyspepsia, and taste disturbance [48]. Zonisamide has been associated with reductions in AHI (33%) and ODI (28%), yet neuropsychiatric tolerability may limit uptake, as dysphoria was reported in about 20% of patients during the open-label phase [49]. Overall, while CAIs consistently improve physiological indices in the short term, response heterogeneity and limited longitudinal safety and efficacy data currently restrict their role to carefully selected patients with elevated loop gain, within a phenotype-driven research framework.

### 3.5. Modulators of Arousal Threshold

A low respiratory arousal threshold is a well-recognized non-anatomical OSA endotype that contributes to ventilatory instability and sleep fragmentation. In these patients, respiratory events often terminate prematurely due to cortical arousal, before sufficient chemical drive and upper airway dilator muscle recruitment can restore airway patency, thereby perpetuating cyclical obstruction and recurrent awakenings [4]. Pharmacological strategies aimed at increasing arousal threshold, alone or combined with modulators of upper-airway neuromuscular activity, have therefore been proposed as a theoretically targeted approach to stabilize sleep and breathing, particularly when anatomical collapsibility is not severe. However, the clinical relevance of this strategy remains uncertain, as the available evidence is largely derived from short, physiology-oriented experiments, most commonly single-night, crossover trials, limiting confidence in durability, safety, and real-world effectiveness.

Among sedative agents, trazodone has been one of the most frequently studied. While mechanistic studies suggest that trazodone can increase arousal threshold without clearly impairing upper-airway muscle responsiveness, more recent controlled data indicate that any improvement in OSA severity is, at best, modest and highly context-dependent. In a double-blind crossover study, trazodone produced only a small reduction in mean AHI, apparently mediated by a reduction in N1 sleep, the stage in which arousals occur most frequently, rather than a robust, endotype-specific normalization of respiratory physiology [50]. Taken together, trazodone may modestly influence sleep continuity in selected phenotypes, but current evidence does not support its routine use as an OSA therapy.

Non-benzodiazepine hypnotics (Z-drugs) have likewise been explored. Eszopiclone has shown inconsistent effects on OSA severity across short double-blind crossover trials (1–2 nights), with conflicting findings on AHI reduction that likely reflect differences in cohort selection; notably, exclusion of patients with marked desaturation in some studies may have indirectly enriched for individuals with a higher baseline arousal threshold, complicating interpretation. Across these short studies, significant adverse effects were not prominent, but the absence of harm signals must be interpreted cautiously given the limited exposure [51]. By contrast, zopiclone and temazepam did not improve OSA severity in single-night, controlled crossover trials; zopiclone nonetheless increased arousal threshold, underscoring the recurring disconnect between measurable physiological effects and clinically meaningful reductions in AHI [52]. Similarly, pimavanserin and zolpidem failed to improve OSA severity in controlled single-night crossover studies and were associated with worse subjective sleep quality, further limiting their clinical appeal as adjuncts for OSA management [52].

In contrast, benzodiazepines remain generally discouraged in OSA due to potential depression of upper airway dilator muscle activity and risk of worsening hypoventilation, particularly in patients with high anatomical collapsibility or more severe disease [53]. Consequently, their use is typically restricted to exceptional circumstances and requires careful monitoring.

Overall, pharmacological modulation of arousal threshold remains an investigational, endotype-informed concept rather than an established therapeutic option. Although certain agents can measurably raise arousal threshold, the current clinical trial evidence, predominantly single-night studies with heterogeneous eligibility criteria, shows inconsistent or absent reductions in OSA severity and, in some cases, deterioration in subjective sleep quality. In particular, sedative strategies warrant heightened caution in patients with severe anatomical obstruction or high collapsibility, where increasing arousal threshold may prolong obstructive events and exacerbate hypoxemia. As such, these drugs should not be framed as routine treatments for OSA; at most, they may warrant further evaluation within carefully phenotyped cohorts (e.g., low arousal threshold, relatively preserved anatomy, prominent sleep fragmentation, COMISA) and within longer, outcomes-focused randomized trials to determine whether short-term physiological signals translate into durable patient benefit [54].

### 3.6. Other Experimental Compounds

Beyond the established classes, several other experimental compounds are being investigated for their potential in OSA treatment. Intranasal administration of leptin in mice has been shown to attenuate sleep-disordered breathing, independent of any body weight reduction [29]. Thyrotropin-releasing hormone (TRH) and its analogs, such as taltirelin, are being explored as potential targets for stimulating the hypoglossal motor nucleus and genioglossus muscle during sleep. In a rat model, taltirelin produced a more prolonged increase in genioglossus motor activity than TRH, making it a promising investigational product [29]. Gene therapy, using designer receptors exclusively activated by designer drugs, has shown remarkable potential in animal models [40]. By injecting an adeno-associated virus carrying an excitatory designer receptor into the genioglossus muscle of mice, researchers achieved a more than six-fold increase in genioglossus tonic electromyographic activity upon activation with a selective agonist [40]. This cutting-edge approach represents a highly targeted attempt to treat OSA pharmacologically and holds significant promise for the future. Importantly, most of these strategies remain preclinical, and their translational relevance is currently limited by the absence of human efficacy and long-term safety data.

### 3.7. Drug Therapy for Residual Excessive Daytime Sleepiness in OSA

EDS is a key symptom of OSA, often prompting patients to seek medical attention. In most cases, positive CPAP therapy is effective in alleviating EDS [55]. However, a subset of patients continues to experience residual EDS (rEDS) despite adherence to CPAP treatment. The prevalence of rEDS, defined by an Epworth Sleepiness Scale (ESS) score > 10, has been reported in about 15% of patients [56]. In population-based studies, rEDS persists in approximately 9–22% of CPAP-adherent individuals [57,58].

The underlying causes of rEDS are multi-factorial [59,60]. A “sleepy” phenotype of OSA has been identified, which is associated with a higher cardiovascular risk [61]. EDS also negatively affects cognitive functioning, productivity, and quality of life [57,62]. Furthermore, it increases the risk of traffic and workplace accidents [63] and is linked to higher rates of depression and anxiety [64]. The most studied WPA for the treatment of rEDS in OSA patients are modafinil/armodafinil, pitolisant, and solriamfetol.

Modafinil and its R enantiomer armodafinil promote wakefulness primarily by inhibiting the dopamine transporter, increasing dopamine signaling in key arousal related brain regions. Modafinil also modulates other wake-promoting systems, including noradrenergic, histaminergic, and orexinergic pathways. Compared with amphetamine like stimulants, it has a lower abuse potential and fewer peripheral sympathomimetic effects, likely due to a more selective and regionally restricted dopaminergic action with limited engagement of reward circuitry [65,66,67,68]. Modafinil was approved by FDA for narcolepsy and for rEDS. In 2016 Kuan and colleagues conducted a systematic review and meta-analysis of randomized controlled trials examining modafinil and armodafinil, in adults with OSA [69]. Meta-analytic pooling showed that modafinil reduced the ESS by approximately three points relative to placebo (weighted mean difference −2.96, 95% CI −3.73 to −2.19), while armodafinil achieved a similar reduction of −2.63 points (95% CI −3.40 to −1.85). Correspondingly, sleep latency on the Maintenance Wakefulness Test increased by about 2.5 min with modafinil and 2.7 min with armodafinil. Functional outcomes improved modestly; one pooled analysis of three trials [70,71] showed that modafinil increased the total score on the Functional Outcomes of Sleep Questionnaire by a mean of 1.28 points, and a sub-analysis indicated benefits in activity, productivity, intimacy and vigilance. These results are clinically relevant in some patients, but the magnitude of change is generally modest and often close to the lower limit of importance in the ESS reduction. As a consequence, many patients improve without fully normalizing symptoms, and improvements in neurocognitive outcomes are less consistent than improvements in subjective sleepiness, with mixed results across domains and study designs [71,72,73]. In addition, controversies persist because postmarketing surveillance and regulatory reviews have raised concerns about cardiovascular adverse reactions, including hypertension and arrhythmias, contributing to restrictions of modafinil indications for OSA in Europe. Longer exposure data remain limited; in a 12 month open label extension, armodafinil was associated with modest average increases in blood pressure and heart rate [74]. Overall, modafinil and armodafinil can be useful for selected patients with persistent rEDS, but the benefit risk balance should be individualized, with particular caution in those with uncontrolled hypertension, arrhythmia history, or broader cardiometabolic vulnerability.

Pitolisant is a selective histamine H3 receptor antagonist and inverse agonist approved for treating symptoms of excessive daytime sleepiness in narcolepsy and OSA [75]. Daily dosage ranges from 9 to 36 mg per day, in a once daily administration in the morning, with a recommended dosage range of 9 to 36 mg per day. The half-life of the drug is 10–12 h, with a peak in plasma concentration at 3 h [76]. Pitolisant pharmacokinetics result in reduced plasma levels by the end of the day, minimizing its wake-promoting effects at night. The most common adverse events with pitolisant included epigastralgia and abdominal pain, increased appetite and weight gain, headache, insomnia and anxiety [77]. RCTs reported a low adverse events incidence, mainly consisting in headache, insomnia, nausea, vertigo, and anxiety, with no cardiovascular or other significant safety concerns [78,79]. The HAROSA-I study was conducted in patients with moderate-to-severe OSA and rEDS despite good adherence to CPAP therapy [80]. The trial aimed to determine whether adjunctive pitolisant (up to 20 mg/day) is effective and overall safe for EDS. Pitolisant produced a mean ESS decrease of −2.6 points versus placebo and improved also objective measures of residual EDS, with a good tolerability profile. The HAROSA-II trial was conducted for patients with OSA who refused or are nonadherent to CPAP therapy, complaining of EDS, yielded similar results [79]. The study included 268 patients who were randomized (200 to pitolisant 20 mg/day and 68 to placebo). In a 1-year open-label extension of the HAROSA-1 and HAROSA-2 randomized trials, pitolisant (up to 20 mg/day) demonstrated sustained efficacy in reducing residual excessive daytime sleepiness in adults with OSA, irrespective of CPAP adherence, with a mean ESS reduction of approximately eight points at 52 weeks. Long-term treatment was well tolerated, including cardiovascular safety, with no new safety signals emerging over one year of follow-up [81]. More recently, the HAROSA-III trial analyzed the impact of pitolisant at higher doses (up to 40 mg once daily) to treat EDS in patients with OSA [79]. The study aimed to assess the efficacy and safety of pitolisant, in patients with OSA, whether they were adherent to CPAP or not. At 12-week pitolisant significantly improved EDS, vigilance, and global impressions in patients with moderate-to-severe OSA, regardless their use of CPAP. The HAROSA studies globally concluded that pitolisant 20 mg and 40 mg reduced EDS and improved quality of life in patients with OSA with residual EDS, regardless CPAP therapy adherence [82].

Solriamfetol is an oral selective dopamine and norepinephrine reuptake inhibitor approved by to promote wakefulness in adults with EDS in OSA and narcolepsy [83]. Several RCTs evaluated the efficacy and safety and tolerability profile of solriamfetol in treatment of EDS in OSA, in both adherent and non-adherent patients [84]. In the TONES 3 trial, a randomized, double-blind, placebo-controlled, parallel-group study, adults with OSA and rEDS were assigned to receive solriamfetol at doses of 37.5 mg, 75 mg, 150 mg, or 300 mg once daily, or placebo, over a 12-week period [85]. At doses of 75 mg and above, solriamfetol significantly prolonged wakefulness and reduced subjective sleepiness, with the 300 mg dose showing the greatest effect. Adverse events were generally mild to moderate and included headache, nausea, decreased appetite, and anxiety. No significant changes were observed in nightly use or adherence to primary OSA therapy across treatment groups. The improvement of EDS was independent by the adherence to primary OSA therapy. Importantly, solriamfetol did not negatively impact ongoing use of OSA therapy [85]. Subsequently, the TONES 4 study demonstrated that continued solriamfetol treatment significantly improved wakefulness compared to placebo, supporting its sustained efficacy in this population [86]. Another recent study (SHARP) focused on the impact of solriamfetol on patients with OSA and cognitive impairment. The SHARP trial demonstrated that two weeks of daily solriamfetol (up to 150 mg) significantly enhances both objective and subjective measures of cognitive functioning in patients with OSA-related EDS and cognitive impairment, with effects sustained up to eight hours post-dose and an acceptable tolerability profile [87]. The most common adverse events with solriamfetol are headache, nausea, decreased appetite, anxiety, and nasopharyngitis [78].

To summarize, all three WPA currently available for the treatment of EDS in OSA (modafinil/armodafinil, solriamfetol and pitolisant) have demonstrated significant efficacy compared with placebo and represent valuable options to address a highly prevalent and debilitating residual symptoms with a major impact on quality of life. This is supported by a recent large network meta-analysis including more than 20 randomized controlled trials and over 4000 patients with OSA, which showed that all agents produced clinically meaningful improvements in both subjective and objective measures of wakefulness, with broadly comparable efficacy across drugs [88]. However, the typical ESS benefit is often in the two-to-three-point range, neurocognitive improvements are less consistent than sleepiness improvements, and the appropriate choice depends on an individualized benefit risk evaluation. A comparative summary of the main wake-promoting agents is provided in Table 1. Modafinil and armodafinil warrant particular caution in patients with hypertension or cardiometabolic comorbidity given ongoing postmarketing cardiovascular safety concerns and the need for monitoring. Pitolisant is a valuable option but requires attention to insomnia, weight gain, and careful titration. Solriamfetol offers robust wakefulness effects but needs blood pressure and heart rate monitoring and thoughtful selection in higher risk patients. None of these therapies replaces primary OSA treatments and they should not be presented as alternatives to CPAP therapy, which remains the cornerstone of disease modifying management.

## 4. Future Directions and Challenges

OSA exhibits heterogeneous pathophysiology, including anatomical upper airway collapsibility, impaired dilator muscle responsiveness, a low respiratory arousal threshold, and ventilatory control instability, which together generate distinct endotypes and variable clinical presentations across patients. While CPAP remains the first line therapy for moderate-to-severe disease, suboptimal adherence frequently limits real world effectiveness and may leave patients with persistent symptoms. In parallel, non-CPAP therapies are playing an increasingly important role within personalized management pathways, and ongoing trials continue to evaluate pharmacological strategies that target specific physiological traits, potentially expanding future options beyond airway splinting alone [33] (see Table 2). Non-CPAP therapies play an increasingly important role in the personalized management of OSA. Future work should focus on phenotype-driven strategies and long-term outcome data [10].

Endotype-based pharmacotherapy remains conceptually attractive, yet its translation into routine care faces substantial implementation constraints. The physiological traits used to define OSA endotypes are most reliably derived from full polysomnography with dedicated trait estimation methods, which are not routinely available in most clinical settings. This limits scalability and introduces variability related to infrastructure, technical expertise, and analytic pipelines, with clear implications for cost and equitable access. Even where advanced analyses are feasible, uncertainty remains regarding trait stability over time and across clinical contexts, particularly with changes in weight, comorbidities, and treatment exposure.

Drug-induced sleep endoscopy can provide useful information on patterns of upper airway collapse and may support selection of anatomical interventions, but it should be interpreted cautiously as a surrogate for mechanistic endotyping. Sedation alters ventilatory drive and neuromuscular tone, and the collapse pattern observed during the procedure may not reflect the balance of non-anatomical traits such as loop gain or arousal threshold during natural sleep. Consequently, relying on drug-induced sleep endoscopy alone may lead to misclassification, especially when the intended therapy targets ventilatory control instability or neuromuscular responsiveness rather than anatomy.

Several unresolved issues should be addressed before endotype guided pharmacotherapy can be meaningfully integrated into clinical pathways. First, most available trials remain short and physiologically focused, leaving major gaps in evidence on long-term outcomes, including sustained symptom benefit, cardiovascular endpoints, and safety in patients with cardiometabolic comorbidity. Second, given the multi-factorial nature of OSA, a key open question is how best to design and validate rational combination strategies, for example, pairing agents that target distinct traits such as ventilatory control instability and upper airway muscle responsiveness, or combining disease-modifying approaches with symptomatic wake-promoting therapy when appropriate. Third, cost effectiveness will likely determine real-world adoption, particularly if endotyping requires advanced polysomnographic analyses or repeated assessments; future studies should therefore incorporate health economic evaluations alongside patient-centered outcomes and safety monitoring.

In summary, endotype-based pharmacotherapy represents a plausible step toward more individualized OSA care, but broader adoption will depend on accessible phenotyping tools and outcomes-focused evidence that demonstrates durable benefit, safety, and value.

## Figures and Tables

**Table 1:** Wake-promoting agents for residual excessive daytime sleepiness in OSA: mechanisms, efficacy, and key safety considerations.

Agent	Primary Mechanism	Typical Efficacy in OSA rEDS	Practical Strengths	Key Limitations and Safety Considerations
Modafinil, armodafinil	Dopamine transporter inhibition, with downstream effects on other arousal systems	ESS reduction about 2 to 3 points vs. placebo, MWT sleep latency increase about 2 to 3 min [69]	Longest clinical experience, modest improvement in sleepiness and vigilance in selected patients [69,70,71,72,73]	Benefit often modest and neurocognitive gains inconsistent [71,72,73]; postmarketing and regulatory concerns regarding cardiovascular adverse reactions, including hypertension and arrhythmias; caution in patients with cardiometabolic disease, longer term data limited [74]
Pitolisant	Histamine H3 receptor antagonist and inverse agonist	ESS reduction about 2.6 points vs. placebo in HAROSA trials, with sustained benefit in open label follow-up in completers [79,80,81,82]	Generally favorable tolerability in RCTs, minimal sympathomimetic profile, useful option when cardiovascular risk is a concern [78,79,80,81,82]	Insomnia and anxiety can occur; increased appetite and weight gain reported, careful titration is needed to balance efficacy and tolerability [76,77,78,79]
Solriamfetol	Dopamine and norepinephrine reuptake inhibition	Improves wakefulness and reduces subjective sleepiness, dose-related benefits in RCTs [85,86]	Robust wake-promoting effect, does not appear to compromise adherence to primary OSA therapy in trials [85,86]	Dose-related increases in blood pressure and heart rate require monitoring; caution in patients with hypertension or cardiometabolic comorbidity; common adverse events include headache, nausea, decreased appetite, anxiety and nasopharyngitis [78,85,86,87]

**Table 2:** Ongoing Clinical Trials on Pharmacological Therapies for OSA (clinicaltrial.gov).

NCT No.	Objectives	Investigated Drug(s)	Phase	Population	Study Design
NCT06928766	Effects of Eszopiclone and Lemborexant in People With OSA	Eszopiclone, Lemborexant, Placebo	Not Yet Recruiting, Interventional, Phase 2	Obstructive Sleep Apnea	Interventional, Randomized Crossover Assignment
NCT05589792	Acetazolamide on REM OSA	Acetazolamide, Placebo	Active, Not Recruiting, Phase 2	Obstructive Sleep Apnea	Interventional, Randomized Crossover Assignment
NCT05293600	Rescue Pharmacotherapy for OSA	Acetazolamide, Trazodone, Placebo	Recruiting, Phase 2	Obstructive Sleep Apnea	Interventional, randomized crossover assignment
NCT05978505	Reboxetine for Sleep Apnea After ENT Surgery	Reboxetine, Placebo	Recruiting, Phase 2	Obstructive Sleep Apnea	Interventional, randomized, placebo-controlled, double-blind study
NCT06462287	Substance P antagonist (aprepitant) effect on aldosterone in OSA + hypertension	Aprepitant, Placebo	Phase 2, recruiting	OSA with hypertension	Interventional, Randomized crossover assignment
NCT05763329	Single-night lemborexant vs. placebo in moderate–severe OSA with low arousal threshold	Lemborexant (Dayvigo)	Phase 1/2, recruiting	Adults with moderate–severe OSA and low arousal threshold	Interventional, randomized, placebo-controlled, double-blind, crossover trial
NCT05289063	Role of statins in vascular endothelial dysfunction in newly diagnosed OSA	Atorvastatin	Phase 1, recruiting	Newly diagnosed, treatment-naïve OSA adults	Interventional, double-blind placebo-controlled parallel group randomized study
NCT06295562	Pharmacotherapy targeting OSA endotypes (loop gain, tone, arousal)	Atomoxetine + oxybutynin; atomoxetine+ trazodone; venlafaxine; placebo	Phase 4, randomized crossover, 44 subjects per arm	Adult OSA patients	Interventional, randomized, placebo-controlled, double-blind, crossover trial

## Data Availability

The original contributions presented in this study are included in the article. Further inquiries can be directed to the corresponding author(s).

## Abbreviations

The following abbreviations are used in this manuscript:

AHI	Apnea–Hypopnea Index
BMI	Body Mass Index
CAI	Carbonic Anhydrase Inhibitor
COMISA	Co-morbid Insomnia and Sleep Apnea
CPAP	Continuous Positive Airway Pressure
DISE	Drug-Induced Sleep Endoscopy
EDS	Excessive Daytime Sleepiness
ESS	Epworth Sleepiness Scale
ESP	Expansion Sphincter Pharyngoplasty
FDA	Food and Drug Administration
FOSQ	Functional Outcomes of Sleep Questionnaire
GIP	Glucose-Dependent Insulinotropic Polypeptide
GLP-1	Glucagon-Like Peptide-1
HNS	Hypoglossal Nerve Stimulation
MAD	Mandibular Advancement Device
MMA	Maxillo-Mandibular Advancement
ODI	Oxygen Desaturation Index
OSA	Obstructive Sleep Apnea
PAP	Positive Airway Pressure
Pcrit	Pharyngeal Critical Closing Pressure
rEDS	Residual Excessive Daytime Sleepiness
RCT	Randomized Controlled Trial
REM	Rapid Eye Movement Sleep
UPPP	Uvulopalatopharyngoplasty
WPA	Wake-Promoting Agent
